# Advanced Phosphorus–Protein Hybrid Coatings for Fire Safety of Cotton Fabrics, Developed Through the Layer-by-Layer Assembly Technique

**DOI:** 10.3390/polym17070945

**Published:** 2025-03-31

**Authors:** Xuqi Yang, Xiaolu Li, Wenwen Guo, Abbas Mohammadi, Marjan Enetezar Shabestari, Rui Li, Shuyi Zhang, Ehsan Naderi Kalali

**Affiliations:** 1 Department of Safety Engineering, Faculty of Geoscience and Engineering, Southwest Jiaotong University, 111 2nd Ring Rd North Section 1, Jinniu District, Chengdu 610032, China; 2 College of Materials and Chemistry, China Jiliang University, Hangzhou 310018, China; 3 Key Laboratory of Eco-Textiles, Ministry of Education, College of Textile Science and Engineering, Jiangnan University, 1800 Lihu Avenue, Wuxi 214122, China; 4 Department of Chemistry, University of Isfahan, Isfahan 81746-73441, Iran; 5 School of Fire and Safety Engineering, Zigong 643000, China

**Keywords:** bio-based flame retardant, layer-by-layer (LbL) assembly coating process, eco-friendly

## Abstract

An advanced, eco-friendly, and fully bio-based flame retardant (FR) system has been created and applied to the cellulose structure of the cotton fabric through a layer-by-layer coating method. This study examines the flame-retardant mechanism of protein-based and phosphorus-containing coatings to improve fire resistance. During combustion, the phosphate groups (−PO₄^2−^) in phosphorus containing flame retardant layers interact with the amino groups (–NH_2_) of protein, forming ester bonds, which results in the generation of a crosslinked network between the amino groups and the phosphate groups. This structure greatly enhances the thermal stability of the residual char, hence improving fire resistance. Cone calorimeter and flammability tests show significant improvements in fire safety, including lower peak heat release rates, reduced smoke production, and higher char residue, all contributing to better flame-retardant performance. pHRR, THR, and TSP of the flame-retarded cotton fabric demonstrated 25, 54, and 72% reduction, respectively. These findings suggest that LbL-assembled protein–phosphorus-based coatings provide a promising, sustainable solution for creating efficient flame-retardant materials.

## 1. Introduction

Among various natural textiles, cotton fabric, made from biodegradable cellulose, offers numerous benefits, including superior hygroscopicity, softness, comfort, and breathability. Due to its distinctive characteristics, cotton fabric is extensively utilized in various domains such as apparel, furniture, military uniforms, home decor, and industrial applications [1,2,3]. Despite its many advantages, cotton fabric’s primary shortcoming is its flammability. This limitation restricts its suitability for high-performance uses which necessitate fire-resistance. Cotton is composed of chain segments of carbohydrate, making it highly flammable. Upon ignition, it undergoes significant degradation, producing highly combustible volatiles that lead to rapid fire spread and substantial smoke release, thereby heightening the risk of fatalities and severe societal damage [4]. Consequently, there is an urgent need to enhance the flame retardancy of cotton fabric to comply with mandatory flammability standards. Thus, modifying cotton fabric to impart superior flame-retardant properties is essential.

Halogen-containing flame retardants are acknowledged as the most efficacious compounds for enhancing the fire resistance of cotton fabrics [5,6]. Nonetheless, their combustion is accompanied by the release of toxic fumes, including carcinogenic dioxins, which pose severe threats to human health and contribute substantially to environmental pollution. Consequently, numerous countries have decided to prohibit the use of halogenated flame retardants [7,8]. Instead, flame retardants containing nitrogen, phosphorus, silicon, and boron are extensively employed for this purpose [9]. Among these, phosphorus-based flame retardants are particularly noted for their efficacy and low toxicity when applied to the cotton fabrics, as conducted by Nguyen et al.’s investigation [10]. During the combustion process, phosphorus-containing flame retardants (FRs) [8] generate nonvolatile phosphorus-based acids. These acids can esterify and dehydrate decomposed cellulose, leading to the formation of a coherent char residue. This char acts as a barrier, impeding the transfer of heat and oxygen between the condensed and gaseous phases, thereby facilitating the cessation of combustion [11]. Commercialized flame retardants, such as Pyrovatex CP^®^ and PROBAN^®^, incorporate reactive phosphorus or N-CH_2_OH groups synthesized with formaldehyde. Consequently, treated cotton fabrics tend to emit formaldehyde during their use [12]. From an environmental protection and ecological outlook, there is a growing emphasis on substituting harmful, toxic, and halogen-containing flame retardants with environmentally safe alternatives [13]. To this end, various techniques have been employed to develop environmental-friendly flame-retardant coatings for the cotton fabric. On the other hand, there are several methods of applying the flame retardant materials to the cotton fabrics including sol–gel processes [14], layer-by-layer (LbL) assembly [15,16], plasma treatment [17], and polyelectrolyte deposition methods [18].

Due to its convenient processing and the abundance of its components, layer-by-layer (LbL) assembly process widely utilized with various materials [19], such as chitosan/ammonium polyphosphate (APP) [20,21], chitosan/montmorillonite [22], chitosan/phytic acid (PA) [23,24], and protein/PA [25,26]. Utilizing the LbL assembly technique, Liu et al. [27] integrated 3-aminopropyl triethoxysilane, sodium phytate, and chitosan to create a nano-coating that capable cotton fabrics with self-extinguishing properties at a coating load of about 32 wt%. Ammonium polyphosphate-derived intumescing flame retardants have garnered significant attention due to their low toxicity and high efficiency. They are frequently utilized in the preparation of layer-by-layer assembly coatings. In a particular study, Fang et al. [28] treated cotton fabric with chitosan and APP using the LbL assembly method. The results indicated that, by increasing the number of bilayers to 20 or more, significantly reduced the heat release rate to approximately one-fifth of that of untreated cotton fabric. Regardless of the main advantages of the LbL assembly technique, such as high customizability and simplicity, it involves a multistep adsorption process that requires specialized equipment and long-term operations, thus hindering large-scale production. Therefore, LbL assembly technique operation steps needs to be minimized [29]. As mentioned before, natural fiber fabrics are highly flammable. Thus, the development of high-performance flame retardants or advanced flame-retardant technologies is crucial to ensure the safety and reliability of natural polymer-based composites. Layer-by-layer (LbL) assembly offers a promising alternative to traditional additive flame retardants due to its high flame-retardant efficiency, environmental acceptability, and minimal impact on the intrinsic properties of polymers [30]. LbL assembly is versatile, cost-effective, and applicable to various materials, including polyelectrolytes, nanoparticles, and biomolecules. It has been utilized for applications such as gas barriers, antimicrobial coatings, biosensing, charge storage, antireflection, and drug delivery [31]. Recently, it has been applied to design flame retardant coating as well. The LbL method provides several advantages over traditional flame retardant techniques. It constructs flame-retardant multilayer films on the substrate’s surface, directly interfering with combustion and avoiding the challenges of incorporating flame retardants into the substrate, which can adversely affect its mechanical properties [32,33]. Additionally, LbL assembly allows for the fabrication of multilayer films with controllable thickness, composition, and function using simple, versatile, and mild experimental conditions, such as room temperature, atmospheric pressure, and low concentrations of assembly materials (below 1 wt%), making it a cost-effective route for fabricating coatings [34,35]. Consequently, in Qiu et al.’s investigation [31], flame retardant coatings fabricated through the straightforward and eco-friendly layer-by-layer assembly method are particularly significant, as they enhance the flame retardant properties of polymers without altering their intrinsic characteristics.

It is worth noting that the LbL assembly method has been effectively utilized to construct thermally insulating and fire-shielding coatings composed of inorganic nanoparticles or hybrid organic–inorganic systems [36,37]. Since its initial application, significant advancements have been made, resulting in enhanced coating efficiency and, at times, unmatched properties. For instance, in the context of cotton, initial systems struggled to preserve fabric structure post-flammability tests, whereas current systems can now achieve self-extinguishing capabilities while maintaining the integrity of most of the fabric [38]. The range of reagents and substrates has expanded to include various nanoparticles and eco-friendly polyelectrolytes, which have been applied to fabrics, foams, and thin films [39,40,41]. Moreover, in-depth studies on deposition parameters have provided a better understanding of the relationship between coating morphology and final properties [32,42].

This work represents an advanced approach focused on the novel employment of a eco-friendly and bio-based layer-by-layer coatings to fabricate a feasible and efficient structure capable of enhancing the fire safety of cotton fabrics for the indoor use. In the present project, phytic and pea protein was used as positive and negative charge coating with the aim of enhancing the fireproof properties of the cotton fabric in air (characterized by flammability and combustion tests, thermo-stability analysis, and spectroscopy, respectively. More particularly, the pea protein exhibits an intumescent-like and char-forming agent system possessing excellent synergy, in which phytic acid is able of formation phosphoric acid at elevated temperatures, thus promoting the char formation. Moreover, pea protein can generate water vapor during the dehydration in the presence of phosphoric acid, and its synergism with phytic acid favors the production of effective residual char that can significantly enhance the resistance of the cotton fabric during combustion significantly. Finally, SEM, FTIR, and Raman spectroscopy were conducted in order to characterize the morphological structure of the flame-retardant coating before and after the combustion, and the performance of the flame-retarded cotton fabrics were considered. For this purpose, thermogravimetric and cone calorimetry analysis were tested to analyze the thermolysis and fire-retardant specifications of the flame-retardant treated cotton fabrics. Finally, the mechanical behaviors were characterized by tensile tests.

## 2. Experiment

### 2.1. Materials

Pure cotton fabrics (100%, 220 g/m^2^) was supplied by the Shaoxing Manheng Textiles Company (Shaoxing, China), and used as the substrate. Phytic acid (PA, 50 wt% aqueous solution) were purchased from Shanghai Macklin Biochemical Co., Ltd. (Shanghai, China). Pea protein was purchased from Shanghai Haiwanyile Biotechnology Co., Ltd (Shanghai, China). All reagents were used to prepare 6 wt% phytic acid solution and 1.5 wt% pea protein solution for layer-by-layer deposition using deionized water.

### 2.2. Preparation of Flame-Retardant Solution

Pea protein was dissolved in 60 °C deionized (DI) water and adjusted the pH value to 9 using the 1 M HCl solution which was stirred for 1 h to prepare 1.5 wt% pea protein solution. A 6 wt% PA solution was prepared by diluting the concentrated PA solution in DI water, and its pH was adjusted to 4 using the 1 M NaOH solution.

### 2.3. Treatment of Pure Cotton Fabrics with LBL Flame Retardant

**Figure 1:**
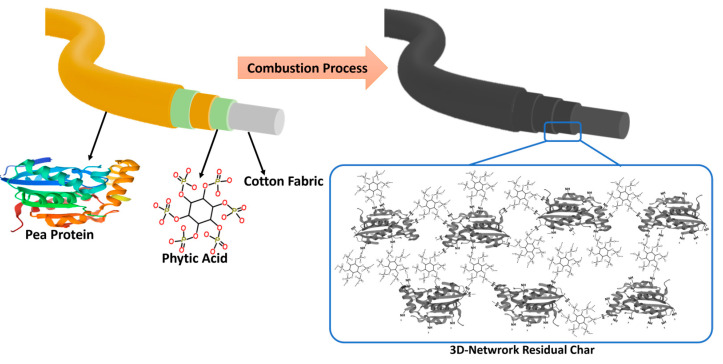
Schematic flame-retardant mechanism of the FR coating. Left: the chemical structure of the flame retardant coating; right: illustration of the network generated during the combustion between the phytic acid and pea protein.

Before the LBL deposition, the cotton fabric was washed with DI water and dried to remove the impurities. The process of preparing cotton fabric flame retardant composite is prepared by alternatively adsorbing the positive and negative polyelectrolyte according to LBL technology, as shown in Figure 1. Cotton fibers usually containing negative charge due to the presence of carboxyl and hydroxyl groups. Therefore, cotton fabric is firstly immersed in the positive pea protein solution for 5 min, then washed with deionized water and dried at 80 °C for 30 min. After that, the cotton fabric was subsequently immersed in the negative PA solution for 5 min, then washed with DI water and dried at 80 °C in the oven for 30 min to complete the 1 bi-layer (BL). The above process was repeated to obtain 3, 6, 9, and 12 BL pea protein/PA flame retardant coating on cotton fabrics which were named by 3BL, 6BL, 9BL, and 12BL.

### 2.4. Characterizations

The surface morphology and elemental distribution on cotton fabrics and residual chars were analyzed using a scanning electron microscope (SEM) (JSM 7800F Prime, OXFROD X-Max 80, Toyama, Japan) at an accelerating voltage of 5 kV, equipped with an energy-dispersive X-ray spectrometer (EDX). To improve electrical conductivity, before microscopy, sputter-coating with chromium under vacuum was applied to the samples.

Fourier-transform infrared (FTIR) spectrums were recorded with a Spectrum 100-T FTIR spectrometer (Thermo Fisher Nicolet iS50, Waltham, MA, USA) across a wavenumber range of 4000 to 500 cm⁻^1^, averaging 16 scans per spectrum at a resolution of 4 cm^−1^. The FTIR spectrometer utilized an attenuated total reflectance method to characterize raw chemicals and cotton fabrics prior and after the coating process.

Thermal stability of the cotton fabric was evaluated using a TGA (Netzsch TG 209 F1, Selb, Germany) instrument with a heating rate of 10 °C/min under nitrogen atmosphere. To ensure accuracy, each specimen was examined twice. The theoretical results were computed using a linear formula of the values from pristine cotton, PA, and pea protein, based on the following equation:

(1) $$W_{th}{\left(T\right)}_{FR}=v\cdot W_{exp}{\left(T\right)}_{PC}+x\cdot W_{exp}{\left(T\right)}_{PA}+y\cdot W_{exp}{\left(T\right)}_{PE},\;v\;+\;x\;+\;y\;=\;1$$

The experimental TG readings of non-treated cotton, PA and pea protein are denoted as $W_{exp}{\left(T\right)}_{PC}$, $W_{exp}{\left(T\right)}_{PA}$, and $W_{exp}{\left(T\right)}_{Pea}$, respectively. The weight percentages of pristine cotton, PA, and pea protein are represented by v, x, and y, respectively. For the above calculation, the weight-gain of each bilayer was approximately 16.5 g/m^2^ and used to calculate the weight of bilayers for each individual specimen. Thermogravimetric analysis was performed at a heating rate of 10 degrees Celsius per minute from 35 °C to 800 °C under a nitrogen atmosphere. About 10 mg of sample was used for this purpose.

Limiting oxygen index (LOI) was determined using a limiting oxygen indexer instrument (HC-2C, Nanjing Shangyuan Analytical Instrument Co., Ltd. Nanjing, China) according to the GB/T 5454-1997 [43] standard regulation, with sample dimensions of 150 mm by 58 mm. The vertical burning analysis was performed employing a vertical burning instrument with sample dimensions of 300 mm by 78 mm, following the GB/T 5455-2014 regulations [44].

A laser-equipped Raman spectrometer (Thermo Scientific DXR, Waltham, MA, USA) was employed to record spectra in the range of 500 to 2000 cm⁻^1^ with a wavelength excitation of 532.17 nm.

Flammability tests were performed using a Cone calorimeter (FTT, Derby, UK) according to the ISO 5660-1 standard [45], with a heat-flux of 25 kW/m^2^ and sample dimensions of 100 mm by 100 mm by 1 mm.

Mechanical properties of the cotton fabrics were evaluated using an Instron electronic universal testing machine (6025/5800R) following ASTM D-5035-11 standard [46], with sample dimensions of 100 mm by 25 mm. A 1 kN cross-head and a tensile momentum of 300 mm/min were used to determine the tensile properties.

The weight gain of the flame-retardant coating on the cotton was calculated using the following calculation format:

Weight gain% = 100 × (W_2_ − W_1_)/W_1_ (2)

where W_1_ is the weight of the initial cotton and W_2_ is the weight of the treated cotton.

The weight gain of the flame retardant coating summarized in Table 1. Each value is the average of 3 readings per each sample.

## 3. Results and Discussion

### 3.1. Structural and Morphological Characterizations

**Figure 2:**
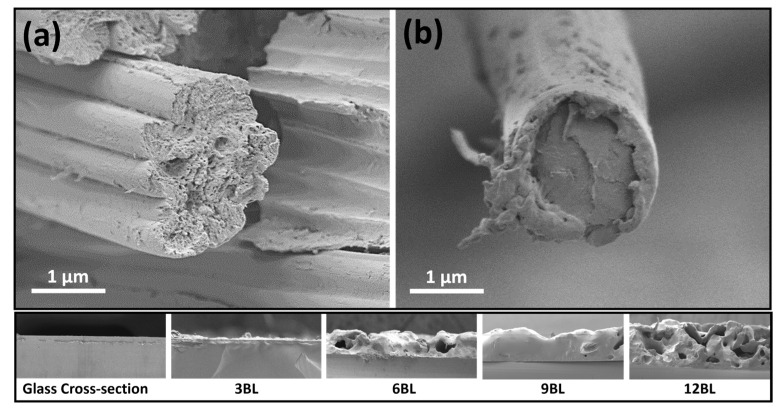
The cotton fibers’ cross section SEM prior (**a**) and after (**b**) coating process, and the micrographs of the cross section of the LbL flame retardants deposited on the silicon wafer.

The formation of the flame-retardant (FR) coating on cotton fabrics was examined using scanning electron microscopy (SEM). SEM images revealed that the applied FR one bilayer coating forms a uniform thin film, (as shown in Figure 2b), surrounding the cotton fibers.

**Figure 3:**
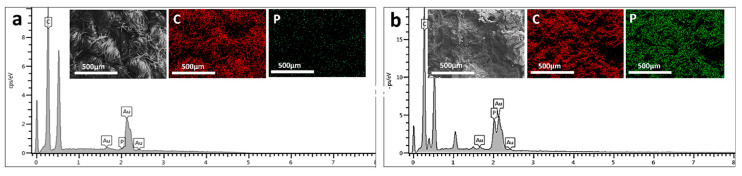
The upper part of the image shows scanning electron microscopy (SEM) micrographs of both untreated and treated cotton fabrics and elemental distribution mapping. The lower part depicts elemental distribution mapping of carbon (C) and phosphorus (P), with panel (**a**) showing the untreated cotton fabric and panel (**b**) showing the treated cotton fabric.

Figure 3 shows that the untreated fabric contains relatively low phosphorus element while after treatment, the treated fabric exhibited an outstanding phosphorus distribution, suggesting successful modification. The elemental spectra additionally validate the higher phosphorus content of the treated over the untreated sample indicative of its increased fire-retardant potential.

**Figure 4:**
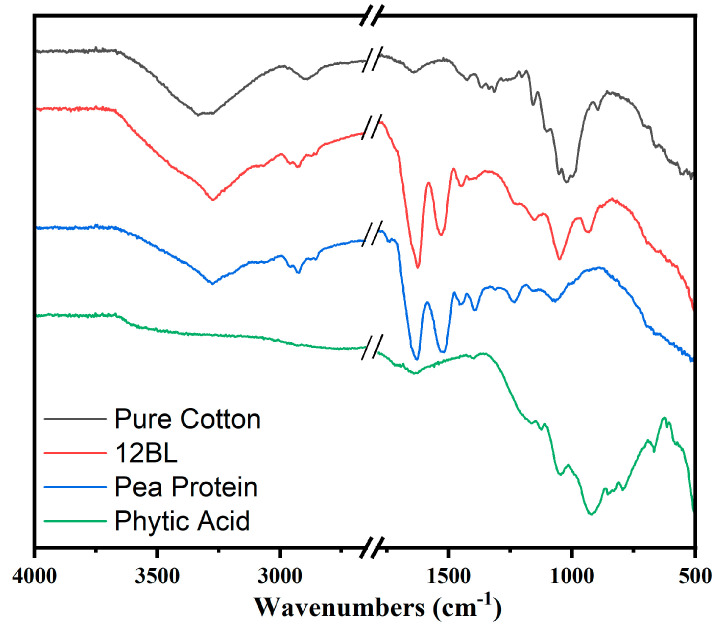
Representative FTIR spectra of pure cotton fabric, and flame retardant coated (12BL) in the presence of pea protein and phytic acid.

The Fourier Transform Infrared (FTIR) spectra (Figure 4) presented in the image compare the characteristic absorption bands of pure cotton, pea protein, phytic acid, and the flame-retardant-coated cotton with 12 bilayers (12BL). The pure cotton spectrum (black line) shows typical cellulose peaks, including a broad absorption band around 3340 cm⁻^1^, which corresponds to O-H stretching vibrations, and a peak near 2900 cm⁻^1^ attributed to C-H stretching. Additionally, the peaks at 1420 cm⁻^1^ and 1370 cm⁻^1^ are associated with C-H bending in cellulose, while the band near 1060 cm⁻^1^ corresponds to the C-O stretching in cellulose. In the spectrum of phytic acid (green line), the characteristic P=O stretching band appears around 1250 cm⁻^1^, and the peak at 900 cm⁻^1^ is indicative of P-O stretching, confirming the presence of phosphorus-based flame retardants. The pea protein spectrum (blue line) shows prominent peaks around 1650 cm⁻^1^ and 1540 cm⁻^1^, which correspond to the amide I and amide II bands, respectively, indicating protein structures. SEM micrographs and elemental mapping of carbon (C) and phosphorus (P) for untreated (a) and treated (b) cotton fabrics are shown in the image.

The 12BL spectrum (red line) reflects the integration of the pea protein and phytic acid with the cotton. The peaks from both phytic acid and pea protein can be seen, with the characteristic peaks of cellulose still present, but slightly shifted or reduced in intensity due to the coating. For instance, the P=O stretching at 1250 cm⁻^1^ and the amide I and II bands from the protein indicate the successful deposition of the flame-retardant bilayers on the cotton fabric. This shift and reduction in intensity signify effective interaction between the cotton fabric and the flame-retardant layers.

### 3.2. Thermal Stability

**Figure 5:**
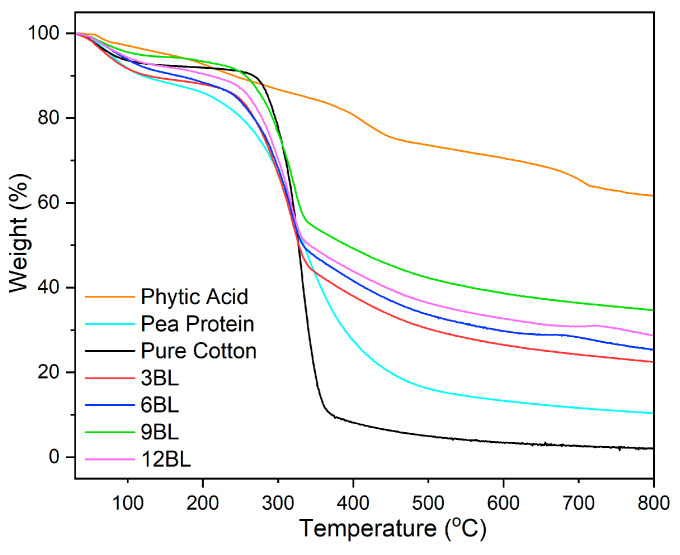
Thermogravimetric analysis of pure and flame retardant coated cotton fabrics.

Generation of the char from decomposed products are critical factors influencing the flame-retardant properties of polymer-based substances. Therefore, it is crucial to study the thermal resistivity of polymer composites. The thermal gravimetric analysis (TGA) data for pristine cotton and its air-free flame-retardant (FR) coatings are outlined in Figure 5 and Table 2. The parameter T_5%_ denotes the temperature at which 5% of the mass is lost and serves as an indicator of thermal stability.

Pristine cotton exhibits a primary degradation step occurring between 240 °C and 450 °C, with a T_5%_ value of 80 °C. After the TGA, there was minimal ash residue left for pristine cotton. In contrast, the 3-bilayer composite, which includes the flame-retardant coating, shows a lower T_5%_ compared to pristine cotton. This founding attributed to the lower decomposition temperature of the used coating compositions. However, increasing the incorporation of phytic acid and pea protein in the 6BL, 9BL, and 12BL resulted in higher T_5%_ values compared to the 3BL composite. Despite these increases, the T_5%_ values of the bilayer compounds remain lower than those of pristine cotton. Moreover, the amount of T_5%_ values in 9BL demonstrates that it is in optimum point and to prove this result the 12BL demonstrated decreases.

This reduced thermal stability is attributed to the lower thermal stability of pea protein and phytic acid, which accelerates the decomposition process within the FR coatings. The addition of the number of bilayers significantly enhanced the residual char yield of the composites. At 800 °C, the char residual yields for 3BL, 6BL, 9BL, and 12BL were 22.4%, 25.3%, 34.7%, and 28.8%, respectively, which are substantially higher than the 2.1% residual yield of untreated cotton. To validate the effectiveness of the FR coating, theoretical residual yields were calculated (Table 2). In all FRC composites, the experimental residual yields exceeded the calculated values, indicating synergistic effects between the cotton fabric, phytic acid, pea protein, and number of layered-by-layered coatings that were previously mentioned.

### 3.3. Flammability

UL-94 and LOI analysis are widely used techniques to evaluate the effectiveness of flame-retardant coatings on treated cotton fabrics [38,39]. As indicated in Table 3, the Limiting Oxygen Index (LOI) of the untreated cotton fabric was 19%, demonstrating a severe fire hazard. In contrast, the LOI value of 3BL reached 23%, representing a 26% increment compared to the untreated cotton fabric. This indicates that the coating transformed the fabric from highly flammable to highly flame-retardant, only at three bilayers.

**Figure 6:**
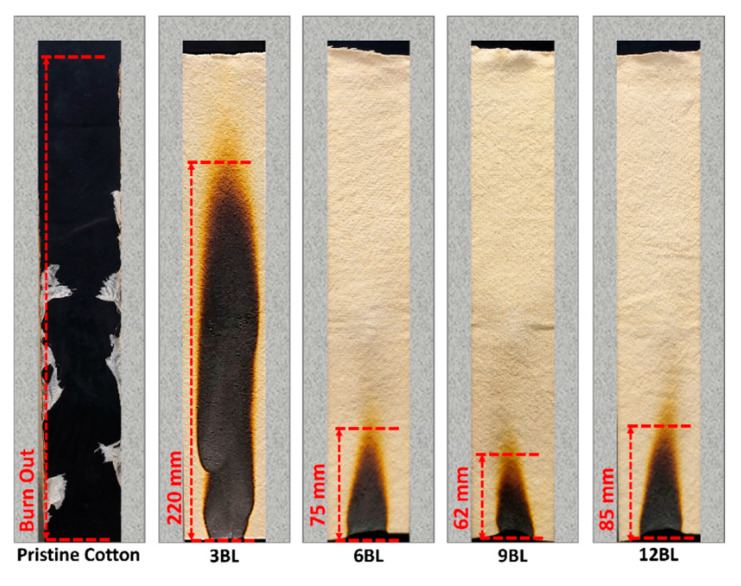
Digital images depicting untreated and fire-retardant-treated cotton fabrics following the vertical burning analysis.

Moreover, according to the digital illustration in Figure 6, the pure cotton fabric immediately ignited upon exposure to fire, with flames rapidly spreading to the top of the sample, resulting in complete destruction. Remarkably, when pristine cotton fabrics were treated with three bilayers, they exhibited an improved ability to form char, resulting in a stable but crumbly residual char; however, it did not pass the UL-94 vertical burning test.

Increasing the bilayers of progressively enhanced the fabric’s resistance to ignition, increased the LOI value, and resulted in formation an intact residual char. Among these, 6BL showed significant difficulty to be ignited, easily secured the vertical burning tests, and achieved an LOI value higher than 3BL. Notably, this pattern is consistent between 9BL and 6BL, with 9BL exhibiting superior performance. However, as previously mentioned, 9BL represents the optimal configuration. Figure 6 further demonstrates that increasing the bilayer to 12BL results in a decline in performance.

From Figure 6, it is evident that less amount cotton fabric was damaged after ignition, and the morphological structural of the char residue remained intact. This indicates that the combination of phytic acid (PA) and pea protein (PE) effectively hindered flame propagation, even at low weight percentages [31,32].

### 3.4. Cone Calorimetry Test

**Figure 7:**
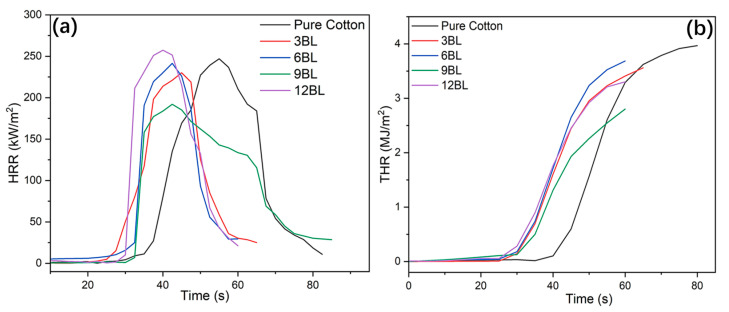
(**a**) HRR and (**b**) THR figures of the pure and flame-retardant treated cotton fabrics collected from the cone calorimetry test.

The fire-resistance properties of untreated cotton and coated cotton fabrics treated with different bilayers (3BL, 6BL, 9BL, and 12BL) of a specific solution were further examined using cone calorimetry. The results, depicted in Figure 7 and summarized in Table 4, include heat release rate (HRR), total heat release rate (THR), and mass-loss data of both untreated cotton and flame-retardant-coated cotton fabric specimens. It is noteworthy that all pHRR values of the flame-retardant cotton samples were significantly lower compared to the untreated cotton control (250 kW/m^2^). The pHRR values exhibited a gradual reduction as the loading of the flame-retardant coating increased. Notably, 9BL demonstrates the lowest pHRR value of 192 kW/m^2^, which is 30% lower than that of untreated cotton. The THRs of the flame-retardant-coated cotton fabrics are significantly lower than those of untreated cotton, reduced from 3.89 to 3.55, 3.64, and 1.82 MJ/m^2^, respectively, indicating an effective suppression of total heat release. The decreased THR suggests that a greater amount of carbonaceous compounds remained in the condensed phase, potentially attributed to the strong synergistic effect of the bilayers’ number. This effect leads to a reduced conversion of combustible organic volatiles into fuel. The inclusion of low percentage of phytic acid and pea protein as the flame-retardant system leads in a substantial increment in formation of residual char, less smoke production, and more fire safety. Formation of residual char and THR were in agreement with the findings from thermogravimetric analysis (TGA). Figure 7 shows that the flame-retardant coated cotton fabrics ignited slightly quicker than untreated cotton because of the rapid decomposition of the flame-retardant coating. The residual char of the flame-retardant-coated cotton fabrics are significantly higher compared to untreated cotton (Table 4). The smoke emission parameters, including the smoke production rate (TSR), total smoke production (TSP), CO production, and CO/CO_2_ proportion are also summarized in Table 4. The TSR and TSP results of the 9BL sample showed significant reduction in comparison with those of untreated and treated cotton fabric.

**Figure 8:**
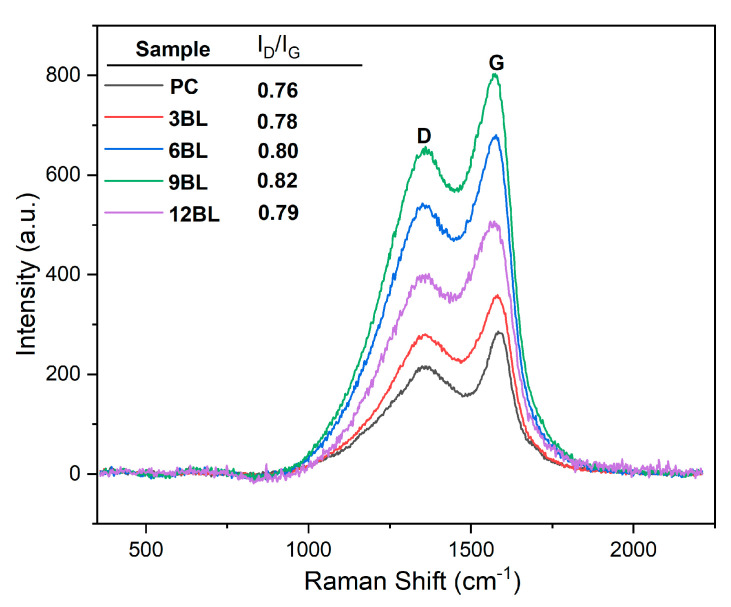
Raman spectra analysis of the residual char of pristine cotton.

Raman spectroscopy is a widely used and effective technique for evaluating the degree of graphitization in residual carbon char, which is closely related to the flame-retardant properties. In this study, Raman spectroscopy (Figure 8) was employed to analyze the char residues of both untreated cotton fabric and the flame-retardant-coated samples. The residual char obtained after the cone tests were utilized for this characterization. The Raman spectra of the char residues exhibited couple of absorption peaks at 1360 cm⁻^1^ and 1568 cm⁻^1^, known as the D and G bands, respectively. The ratio of the integrated intensity of these two bands (I_D_/I_G_) was used to determine the graphitization degree of the residual chars [40]. The D peak (disorder band) indicates the presence of disorder in the carbon planar structure due to defects or functional groups. The G peak (graphite band) arises from the E_2g_ mode of graphite, and involves vibrations of sp^2^-bonded carbon atoms in a 2D-hexagonal lattice.

The base sample has the lowest I_D_/I_G_ ratio, showing less disorder compared to the layer-by-layer flame-retardant-treated samples. As the number of layers increases, the I_D_/I_G_ ratios rise from 0.78 to 0.82 for 3BL to 9BL, indicating more disorder or defect density. For the 12BL sample, a slight decrease in the I_D_/I_G_ ratio to 0.79 suggests stabilization or fewer defects at this processing stage. In general, highly graphitized carbon materials have better thermal stability, meaning they retain their structure at high temperatures.

In flame retardant applications, the formation of a stable char layer is crucial, as it shields the material from further combustion. Such materials can form more stable, less flammable char layers. The 9BL sample, with the highest I_D_/I_G_ ratio (0.82), shows the most disorder, but still maintains good flame retardant properties. The higher D peak in 9BL may aid in better char formation during combustion. For instance, in some studies, alongside an increase in the I_D_/I_G_ ratio, a significant increase in the LOI values is observed, suggesting improved performance due to its structural defects. These defects could promote cross-linking reactions, forming a protective char layer that insulates the material, reduces heat release, and lowers flammability. The strong char formed in 9BL could result from both structural features and flame retardants, creating a more cohesive heat barrier [47,48,49].

**Figure 9:**
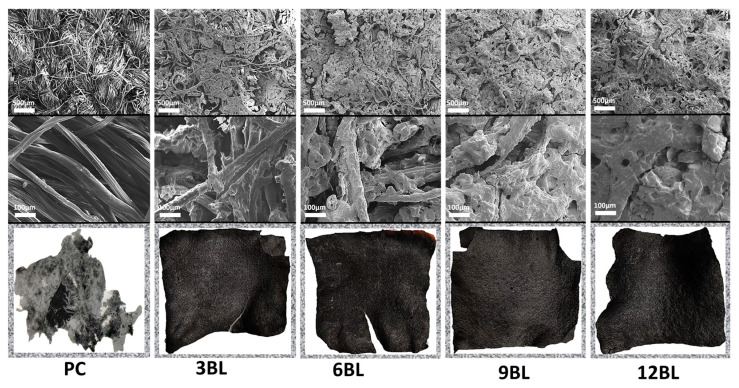
SEM micrographs and digital images of the residual chars for pure cotton, 3BL, 6BL, 9BL, and 12BL after the cone calorimetry test.

To gain a thorough understanding of the flame-retardant mechanism, we conducted SEM and EDX spectroscopy characterization of the residual chars on the flame-retardant coated cotton fabric. Figure 9 presents micrographs of both untreated cotton fabric and flame-retardant-coated specimens after the cone tests at two different amplifications. The observations revealed that in the case of 3BL, a dense and continuous protective layer uniformly covered the surface of the cotton fibers. In contrast, the char residue of the untreated cotton fabric appeared fluffy, fragile, thin, and unstable.

**Figure 10:**
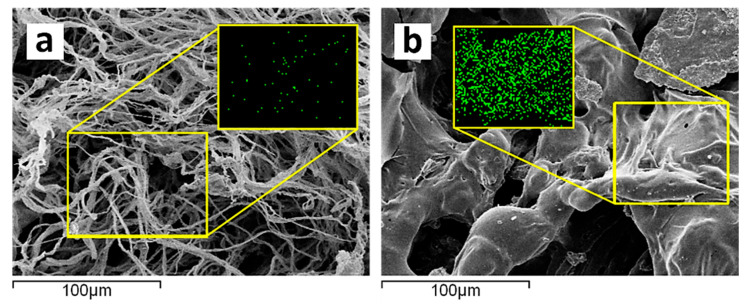
Phosphorus elemental mapping (EDAX) of the residual chars of the pure cotton (PC) (**a**), and 9BL sample (**b**).

Additionally, the weft–warp structure of the FR-coated samples remained intact, similar to the specimen prior the cone test. The EDX spectroscopy results showed a uniform dispersion of phosphorus (P) element within the char layer (Figure 10), indicating the effective action of the flame-retardant coating in promoting condensation. It is hypothesized that phosphoric acids derived from phytic acid could catalyze the cotton fabric bilayers. The combination of these observations suggests that the formed char layer acts as an efficient insulation coating, preventing oxygen and heat transferal to the substrate.

The reaction of phytic acid and pea protein during combustion was considered in order to propose the flame-retardant mechanism of the coatings. The reactions are among the pea protein and the phytic acid, particularly a crosslinking network between amino groups in pea protein and phosphate groups from phytic acid as a dominant reaction (Figure 1). The reaction occurs by phosphorylation of the amino groups (–NH_2_) of the pea protein, and this esterification can result in crosslinking between phosphate groups (–PO_4_^2−^) of phytic acid and the amine groups in the protein.

**Figure 11:**
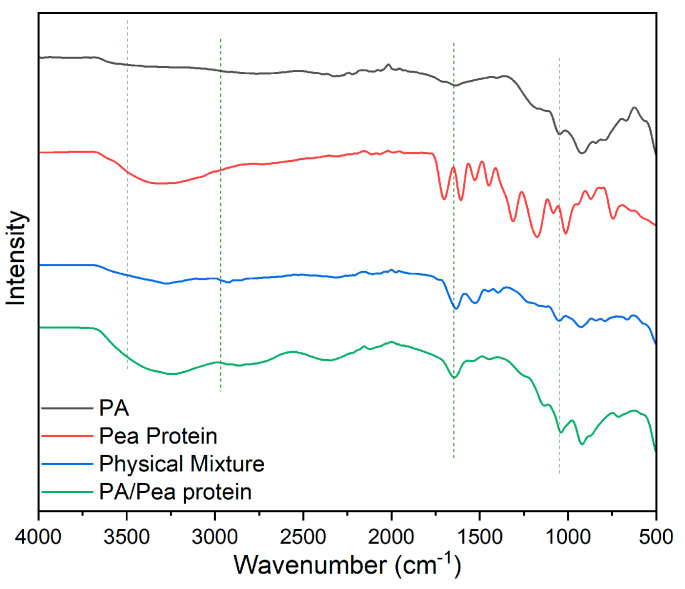
Representative FTIR spectra of the pure FR materials used in this work, and their physical and chemical mixture.

To explain the flame-retardant mechanism of the coatings, the combustion interactions between phytic acid and pea protein were considered. A key reaction between the pea protein and phytic acid involves a crosslinking network formed between the amino groups in the pea protein and the phosphate groups from the phytic acid (Figure 11). This reaction occurs through the phosphorylation of the amino groups (–NH_2_) in the pea protein, and this esterification leads to crosslinking between the phosphate groups (–PO_4_^2−^) from phytic acid and the amine groups in the protein. This type of crosslinking enhances the thermal stability of the char, improving fire retardancy performance [50]. According to the FTIR results shown in Figure 11, the amide I band at 1650 cm⁻^1^, which represents the physical mixture, disappears due to the interaction with the protein’s carbonyl group. Additionally, some peaks below 1000 cm⁻^1^, which correspond to the individual fingerprints of phytic acid and pea protein, merged or changed due to new chemical interactions.

On the other hand, peaks around 1000–1200 cm⁻^1^, associated with the P–O and P=O vibrations, shifted or intensified, indicating an interaction between the phosphate groups and the protein side-chains. Additionally, sharper or more distinct peaks in the 3000–3500 cm⁻^1^ region, related to hydrogen bonding, revealed the formation of stronger hydrogen bonds in the chemical mixture. As a result, the chemical interactions between phytic acid and pea protein (as seen in the FTIR spectra) lead to a uniform chemical blend with strong hydrogen bonds, covalent or ionic phosphate–protein connections, and amide involvement. It can be concluded that these hydrogen bonds help stabilize the structure at lower temperatures. The phosphate groups catalyze the formation of char and promote crosslinking, while the protein’s carbon and nitrogen components contribute to carbonization, forming a nitrogen-doped carbonaceous network. After combustion, the resulting char forms a 3D-network composed of phosphorus-rich and nitrogen-doped carbon structures (Figure 1). This network is thermally stable, mechanically strong, and chemically homogeneous, reflecting the synergistic interactions seen in the FTIR spectra of the chemical mixture [51,52].

### 3.5. Mechanical Properties

**Figure 12:**
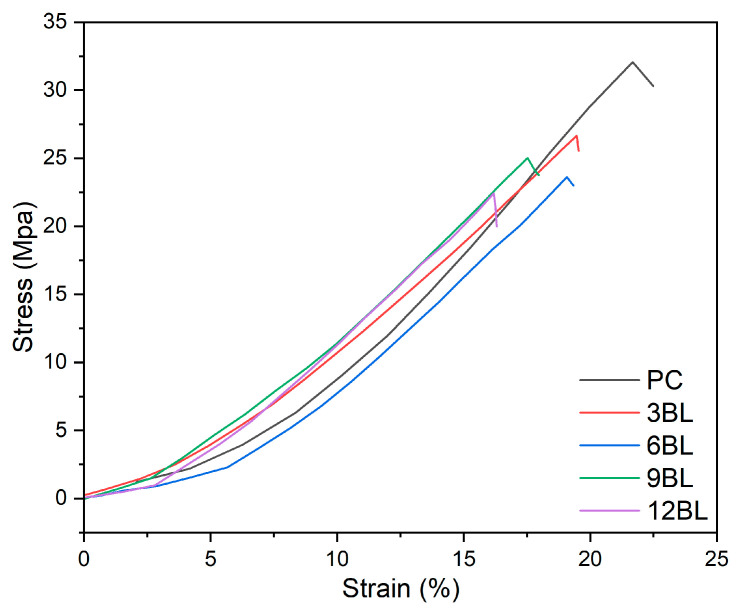
Stress–strain diagrams of pure cotton (PC) and flame-retardant-treated cotton fabrics.

The tensile test results summarized in Figure 12 and Table 5, compared the mechanical behavior of PC fabric with flame-retardant-coated cotton fabrics with 3, 6, 9, and 12 bilayers of flame retardant coatings. As illustrated, the stress–strain curves indicate that pure cotton fabric (PC) reaches an optimal tensile strength of around 32 MPa with a strain of approximately 21%, reflecting better mechanical integrity. In contrast, the flame-retardant-coated fabrics show a decrement in stress and strain as the bilayer count increases. For example, the 3BL fabric shows a similar trend to pure cotton, but with reduced peak strength and stretch. As the number of bilayers increases, especially with 6BL, 9BL, and 12BL fabrics, the stress at failure demonstrates even more drops, with the 12BL fabric displaying the weakest mechanical performance. This suggests that adding more flame retardant bilayers compromises mechanical integrity, possibly due to increased brittleness or stiffness from the coating materials. However, the 12BL fabric still achieves stress levels above 22 MPa, retaining a substantial portion of its strength.

## 4. Conclusions

In this study, we tried to demonstrate the successful development of a sustainable, highly effective flame retardant coating for cotton fabrics using the layer-by-layer assembly technique. By incorporating bio-based materials such as pea protein and phytic acid, this coating enhances the flame resistance of cotton fabrics while maintaining a balance between mechanical integrity and fire protection. The flame retardant coating exhibited great decrement on the flammability of the cotton fabric during the vertical burning test, while showed 70% increment to the LOI values of the 9BL samples compare to the pure cotton fabric. The cone calorimeter results revealed a 30% reduction in the peak heat release rate (pHRR), 46% reduction in the total heat release rate (THR), and 30% reduction in the total smoke production (TSP) for the 9-bilayer (9BL) coated fabric compared to the untreated fabric, while the residual char increased significantly. Thermogravimetric analysis (TGA) further showed a substantial improvement, with the 9BL fabric exhibiting a 34.7% char yield at 800 °C, compared to just 2.1% for pure cotton. While the mechanical properties of the fabrics gradually decrease with the increasing number of bilayers, the 9-bilayer configuration offers an optimal trade-off, delivering substantial flame retardancy without severely compromising strength. These findings highlight the potential of eco-friendly flame retardant coatings to enhance the safety of cotton fabrics, opening new possibilities for their application in industries where fire protection is critical.

## Figures and Tables

**Table 1:** Weight gain percentage.

Number of BL	Pure	3BL	6BL	9BL	12BL
Weight gain (%)	0	29.3	32.2	40.7	47.9

**Table 2:** TGA results.

Sample	T_5%_ (°C)	Residual Yield @ 800 °C (Under N_2_), wt%		Residual Yield @ 800 °C (Under Air), wt%
		Calculated	Experimental	
Phytic acid (PA)	155	--	61.7	5.0
Pea Protein (PE)	79	--	10.3	5.0
Pure Cotton (PC)	80	--	2.1	3.78
3BL	72	10.1	22.4	18.3
6BL	88	13.9	25.3	20.7
9BL	111	16.9	34.7	28.4
12BL	95	19.5	28.8	23.6

**Table 3:** Flammability assessment outcomes for both untreated and treated textiles.

Sample	LOI (%)	Damage Length (mm)	After Flame Time (s)
Pristine Cotton	17	Burn out/damaged char	38
3BL	23	220	15
6BL	26	75	3
9BL	29	62	1
12BL	26	85	3

**Table 4:** The results for both pure and treated cotton fabrics from cone colometry test.

Sample	pHRR (kW/m^2^)	THR (MJ/m^2^)	FIGRA (kW/m^2^⋅s)	Residual Mass (%)	TSR (m^2^/m^2^)	TSP (m^2^)	MARHE (kW/m^2^)	CO (kg/kg)	CO/CO_2_
Pure Cotton	250	3.89	6.25	2.6	42.5	0.60	55.5	0.200	0.0128
3BL	230	3.55	5.85	35.2	68.8	0.58	60.2	0.044	0.0354
6BL	241	3.64	6.42	38.6	67.1	0.62	66.2	0.033	0.0304
9BL	192	1.82	5.02	41.8	13.0	0.17	50.9	0.034	0.0270
12BL	255	3.20	6.80	37.4	44.3	0.49	59.5	0.045	0.0326

**Table 5:** Tensile test results of the pure and flame retarded cotton fabrics.

Sample	Elongation at Break (%)	Stress (MPa)
Pure Cotton	21.6	32.1
3BL	19.4	26.6
6BL	19.0	23.6
9BL	17.5	25.1
12BL	16.1	22.4

## Data Availability

Data are contained within the article.
